# A postal survey of data in general practice on the prevalence of Acquired Brain Injury (ABI) in patients aged 18–65 in one county in the west of Ireland

**DOI:** 10.1186/1471-2296-10-36

**Published:** 2009-05-19

**Authors:** Fionnuala Finnerty, Liam Glynn, Brendan Dineen, Finbarr Colfer, Anne MacFarlane

**Affiliations:** 1 Department of Medical and Social Care Education Medical School, Leicester, LE 1 9HN, UK; 2 Department of General Practice, National University of Ireland, Galway, Galway, Ireland; 3 Acquired Brain Injury Ireland West Region, Galway, Ireland

## Abstract

**Background:**

Very little is known about the prevalence of acquired brain injury (ABI) in Ireland. ABI prevalence has previously been obtained from Belgian general practitioners using a postal survey. We attempted to ascertain the prevalence of ABI in County Mayo through a postal survey of all general practitioners in the county.

The specific objectives of this project were to:

1. identify whether general practitioners are

a. aware of patients with ABI aged 18–65 in their practices

b. able to provide prevalence data on ABI in patients aged 18–65

c. able to provide data on age, gender and patient diagnosis

2. analyse prevalence of ABI from any available data from general practitioners.

**Methods:**

A pilot postal survey was performed initially in order to assess the feasibility of the study. It was established that general practitioners did have the necessary information required to complete the questionnaire. A main postal survey was then undertaken. A postal questionnaire was administered to all general practices in County Mayo in the west of Ireland (n = 59). The response rate was 32.2% (n = 19).

**Results:**

General practitioners who replied on behalf of their practice could provide data on patient age, gender and diagnosis. In the nineteen practices, there were 57 patients with ABI. The age-specific prevalence of ABI in the area surveyed was estimated at 183.7 per 100,000. The mean patient population per practice was 2,833 (SD = 950). There were found to be significantly more patients with ABI in rural areas than urban areas (p = 0.006). There were also significant differences in the ages of patients in the different ABI categories. Patients whose ABI was of traumatic origin were significantly younger than those patients with ABI of haemorrhagic origin (p = 0.002).

**Conclusion:**

Although this is a small-scale study, we have ascertained that general practitioners do have data on patients with ABI. Also, *some *prevalence data now exist where none was available before. These can be used to form the basis of a more substantial programme of university/community partnership research which could provide medical and psychosocial benefits for people with ABI and their families.

## Background

Acquired brain injury (ABI) is a major medical problem in Ireland, other European countries and worldwide [1,2]. ABI has been defined as "*damage to the brain, which occurs after birth and is not related to a congenital or a degenerative disease. These impairments may be temporary or permanent and cause partial or functional disability or psychosocial maladjustment *[3]. It includes traumatic brain injury, haemorrhage, brain tumours, hypoxic brain injury and infection [4]. There are other major epidemiologically significant neurological conditions (e.g. stroke, traumatic brain injury, inter-cerebral haemorrhage, subarachnoid haemorrhage) but our focus on ABI is a result of a community/university partnership between NUI Galway's Department of General Practice and Acquired Brain Injury Ireland (, a non-statutory organisation which was established in 2000 to respond to the diverse needs of people with ABI living in Ireland. This partnership is supported by National University of Ireland, Galway Community Knowledge Initiative: this is a scheme which encourages greater 'civic engagement, linking community and university'.

ABI is a major cause of physical and mental disability in young people. Traumatic injuries, which may result in ABI in those who survive, are the principal cause of death in those aged 15–44 [5]. ABI has significant consequences in people's daily lives: increased aggression, poor memory, concentration difficulties and speech impairments. Such sequelae are known to impact negatively on their personal and social lives and potentially in their ability to work [6].

Although extensive literature exists discussing effective models of care and best practice in brain injury rehabilitation, there is negligible reliable information available on the prevalence of ABI [5,6]. A recent systematic review of studies of brain injury epidemiology in Europe found that good quality prevalence data were unavailable for any European country and that there were critical differences in the methods and definitions of ABI employed by researchers. The development of research guidelines to standardise definitional, case finding and data reporting parameters were recommended in order to establish a more precise description of and hence utility of the epidemiology of brain injury, beyond that of traumatic brain injury only, in Europe [5].

Recent research in Belgium has attempted to ascertain if epidemiological data on ABI in patients aged 18–65 could be gleaned from general practitioners by postal survey as they were well placed in the community to provide this data [2]. This was one of the first attempts to report epidemiological data on the prevalence of ABI in a Western European society in this manner. They estimated the age-specific prevalence of ABI in 18–65 year olds to be 183 per 100,000 [2].

In Ireland, it is estimated that there are approximately 500,000 people with neurological conditions and that annually as many as 13,000 suffer head injuries [7]. According to Hospital In-Patient Enquiry (HIPE) data, over 10,000 people are admitted to Irish hospitals each year with stroke as a primary diagnosis [8]. Furthermore an estimated 30,000 people are survivors of stroke, many of whom have residual disabilities (e.g. hemiparesis, cognitive impairment) following stroke [9]. However, it is important to note that most of the existent information regarding brain injuries is limited to traumatic brain injury (TBI) only [5], which thus underestimates ABI prevalence due to causes other than trauma.

Efforts towards determining ABI prevalence in Ireland – based on extrapolating data from other settings – have been carried out. However, such attempts have been problematic mainly due to differences in the case definitions used and in terms of the respective population compositions being considered. Central to this predicament is the absence of a regional or national reporting system/register of persons with ABI. This has hindered appropriate service development and delivery for patients with complex health and social needs. Currently, the majority of people with ABI are discharged from hospital care where there are limited community rehabilitation services (e.g. physiotherapy, occupational therapy, speech and language therapy). This is particularly the case for patients aged between 18 and 65 years who are not eligible for children's or older people's services [6]. Anecdotal evidence suggests that difficulties which arise for people with ABI and their families are dealt with by general practitioners [6]. It was thus decided to conduct a prevalence study of ABI through general practices in one county in the west of Ireland in order to estimate the prevalence of ABI in the catchment area.

The specific objectives of this project were to:

1. 1 identify whether general practitioners are

a. aware of patients with ABI aged 18–65 in their practices

b. able to provide prevalence data on ABI in patients aged 18–65

c. able to provide data on age, gender and patient diagnosis

2. analyse prevalence of ABI from any available data from general practitioners

## Methods

### • Designing the questionnaire and project materials

The questionnaire consisted of one page asking the practice name, the number of general practitioners working in the practice, and the practice patient population. Next a filter question enquired as to whether the general practitioners were aware if there were any ABI patients in their practice population.

They were then further asked to subdivide these patients into different categories depending on the type of ABI acquired and to provide information on the patient's age, gender and age at diagnosis (See Additional File 1).

General practitioners also received a personalised letter outlining the study aim, a consent form and an information leaflet regarding ABI. The information leaflet was drafted in conjunction with the Acquired Brain Injury Ireland organisation, to ensure that the information provided about the condition was correct. Their input was invaluable because, in our partnership, they had the most expertise about ABI. Therefore, they could check and verify the accuracy and relevance of material in the research information leaflet. An information leaflet about the services provided by Acquired Brain Injury Ireland for patients with ABI aged 18–65 was also provided.

### • Collection of names and addresses

Names and addresses for general practices in County Mayo were obtained from the Health Services Executive Primary Care Unit, Galway. There were 59 general practices in total.

### • Sending the questionnaire

The questionnaire, information leaflet, consent form, personalised letter and prepaid envelope for the return of questionnaire and consent form were sent out in late July. A reminder letter was sent out ten days later to non-respondents and follow-up phone calls were made one week later to those general practitioners who had not yet returned their questionnaires. If the general practitioner was not available to take the phone call, another phone call was made at a later time and if this proved unsuccessful a message was then left with the secretary. The offer was made to resend the questionnaire to any general practitioners who had mislaid their copy. Six general practitioners requested to be resent the questionnaire. Any general practitioners who were on holidays were contacted again in the last week of August.

The Dillman criteria for timing of questionnaire reminders had to be modified because of the short time scale but we used all possible criteria to increase the return of questionnaires – based on a systematic review of questionnaire methods – that included reminder letters, follow-up phone calls and personalised letters [10,11]. On follow-up phone calls, two general practitioners (3.3%) declined participation in the study and five general practitioners (8.2%) said they had received a considerable number of questionnaires over the past few months and were extremely busy.

Ethical approval was obtained from the research ethics committee of the Irish College of General Practitioners.

### • Analysis of the data

Calculation of the point prevalence for ABI was undertaken based on the number of known reported ABI cases within the estimated age-specific patient population of those aged 18–65 years. The 95% confidence interval for ABI prevalence was also determined. Both descriptive (frequencies, means, standard deviations) and inferential statistical analyses (Pearson's χ^2 ^and one-way ANOVA) were performed in SPSS for Windows (version 14.0).

## Results

### General practices

Overall, 59 general practices received the questionnaire. Nineteen practices (response rate of 32.2%) replied to the survey. The main reasons documented for non-response during fieldwork were that general practitioners were too busy to take part and received too many requests to take part in research projects.

Of our respondents, 57% were rural and 38% were single-handed. Sixteen practices indicated they knew of persons with ABI in their practice population while two stated there were no cases of ABI affiliated with their practice. One further practice indicated not knowing whether there was someone with ABI in their patient population.

The total estimated patient population reported by the 19 respondent GPs was approximately 50,360. The mean patient population per practice was 2,833 (SD = 950); the median practice size was 3,000. According to 2006 Census data, 61.64% of the County Mayo was aged between 18 and 65 years [12]. Applying this percentage to the reported estimated patient population, within the respondent practices, there were approximately 31,030 persons of the target age group (18–65 years) in the practices that participated in the survey.

### Subjects with acquired brain injury

**Figure 1:**
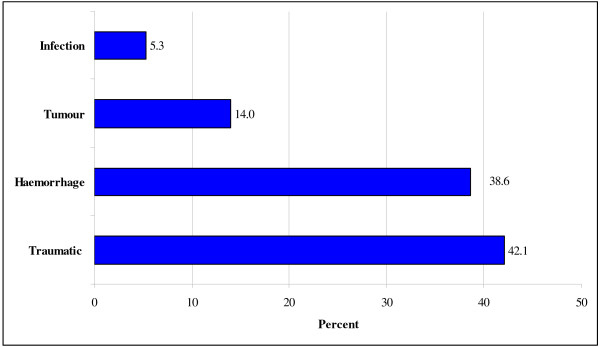
**Percentile distribution of ABI cases by aetiology of injury (n = 57)**.

A total of 57 patients with ABI were reported to be known to the practices. The number of known ABI cases per practice ranged from one to eleven in the 16 practices that reported cases. The overall distribution of ABI cases for all the practices is presented in Table 1. ABI injury according to aetiology is shown in Figure 1.

**Table 1:** Distribution of the number of ABI cases per GP surgery

**Number of ABI cases**	**Number of surgeries with** **this number of cases**
0 *	3
1	4
2	2
3	5
4	1
6	2
7	1
11	1

Total	19

Note: Corresponds to two 'No known cases' and one 'Don't know' response.

Based on the estimated population aged 18–65 years in the respondent practices (31,030), the age-specific prevalence of ABI was estimated to be 183.7 per 100,000. The 95% confidence interval (CI) for the prevalence of ABI is 139.2 to 237.9 per 100,000.

### Gender

For all ABI types except tumour (Table 2), males appeared to be over-represented though this difference was not statistically significant (Pearson χ^2 ^test p = 0.31).

**Table 2:** Distribution of ABI type by gender (p = 0.31)

**Type of ABI**	**Male** **n (%)**	**Female** **n (%)**
Traumatic	20 (48.8)	4 (26.7)
Haemorrhage	15 (36.6)	6 (40.0)
Tumour	4 (9.7)	4 (26.7)
Infection	2 (4.9)	1 (6.6)

Total	41	15

Note: Details regarding the gender of one case of ABI due to haemorrhage were not provided.

### Location

Twenty-six (45.6%) patients attended a general practice located in urban settings, with the remaining 31 persons with ABI (54.4%) being noted by general practitioners from rural areas. Pearson χ^2 ^testing revealed statistically significant differences in ABI type according to patient location (p = .006) as shown in Table 3. Furthermore, when comparing traumatic versus all other aetiologies combined, those patients with a traumatic ABI are significantly more likely to reside in a rural area (p = .008).

**Table 3:** Distribution of ABI type by location (p = .006)

**Type of ABI**	**Rural** **n (%)**	**Urban** **n (%)**	**Total** **n (%)**
Traumatic	18 (58.0)	6 (23.1)	24 (42.1)
Haemorrhage	6 (19.4)	16 (61.5)	22 (28.6)
Tumour	6 (19.4)	2 (7.7)	8 (14.0)
Infection	1 (3.2)	2 (7.7)	3 (5.3)

Total	31	26	57

### Age

The mean age for patients with ABI was 44.5 years (SD = 13.4; 95% CI: 40.9 – 48.1) whereas the mean age at being diagnosed with ABI was 37.21 years (SD = 15.5; 95% CI: 33.1 – 41.3). Four persons incurred their brain injury at young ages (4, 7, 14 and 17 years) while the remaining cases occurred in persons at least 18 years of age.

Table 4 illustrates mean age and SD according to ABI type. A one way ANOVA analysis demonstrated that these differences were statistically significant (F = 5.71, p = 0.002) with youngest mean age associated with an infective cause, followed by trauma, then by tumour and finally by haemorrhage. Post-hoc analysis (Scheffe's test) indicated a statistically significant difference in mean age difference between traumatic and haemorrhage-related ABIs. No other inter-group differences across the four ABI types were statistically significant.

**Table 4:** Distribution of the mean age at time of diagnosis by type of ABI (n = 57) (p = 0.002)

**Type of ABI**	**Mean Age at diagnosis**	**Standard Deviation**
Infection	27.33	6.028
Traumatic	30.04	12.124
Tumour	38.00	20.078
Haemorrhage	46.09	13.473

## Discussion

The purpose of the study was to conduct a prevalence study of ABI through general practices in County Mayo. Key findings are that general practitioners are in possession of the information that was sought and are able to identify patients with ABI in their respective practices. The age-specific prevalence of ABI in County Mayo for those 18 to 65 years was 183.7 per 100,000 (95%CI: 139.2–237.9 per 100,000). Upon extrapolating the age-specific ABI prevalence obtained in this study to the target population of all 18–65 year olds in County Mayo (76,300), the estimated magnitude of ABI is approximately 140 persons (95% CI: 106 – 182) in this age group. ABI was found to be more common in males, to be principally due to trauma, and to be more prevalent in rural areas. These findings reflect a similar epidemiological profile to that reported in previous studies [1,2].

Traumatic brain injuries and tumours were more common in rural areas whilst haemorrhage and infection were noted more in urban settings. Age at diagnosis and the current age of patient were statistically different between the different categories of ABI. Patients whose ABI was of infectious origin were significantly younger than the other groups, with a mean age of 27.33 (SD = 6.028).

However, the data collected must be interpreted with some degree of reservation for two reasons. Firstly, some patients with ABI do not attend their general practitioner and there could possibly be under-reporting of subtler neuro-psychological-related ABI [13,2]. Secondly, the findings may be affected by non-response bias, given the low response rate (32.2%) from the 59 practices. Response rates to postal surveys by general practitioners are often low and this has been attributed to the sheer volume of questionnaires being received regularly by general practitioners and sometimes to the characteristics of the general practitioners themselves [14,15]. In our research, all attempts were made to maximise response rate in the short time allotted for the study. However, many potential respondents said that they did not have time to take part in the survey and emphasised that they do receive many requests to take part in research projects. Also, the fact that the study was conducted in July and August – the traditional summer holidaying months – may have been another contributing factor to the low response.

Few studies have been carried out concerning the epidemiology, incidence and prevalence of ABI. Such studies have differed significantly in the definition of ABI, the ages involved and the reporting methods [5]. We have attempted to ascertain the age-specific prevalence of ABI in one county in Ireland by using general practitioner postal questionnaires. Although the general practitioners could furnish us with the information and the data that was required, the response rate was notably low. As such it is possible that this study may have underestimated ABI prevalence within this population. Further research on this condition would need to utilise additional methods to improve response rates or alternative methods of data collection (e.g. capture-recapture techniques [16,17]. Finally, concerted efforts should be made to establish a dedicated ABI register in order to facilitate access to social and health service provision by those with ABI.

One of the most positive aspects of the study was the strengthening of a previous academic link between the Acquired Brain Injury Ireland organisation and the Department of General Practice, NUIG which can be further developed into future programmes of university/community partnership research about ABI.

## Conclusion

Acquired brain injury is a very large problem worldwide. These patients often lack access to adequate services. In Ireland, no reliable prevalence data for patients with ABI exist, with figures being extrapolated from abroad. General practitioners do possess the relevant information with regard to patients with ABI in their practices and can provide prevalence data, despite certain limitations. This present study identified an age-specific prevalence comparable to at least one other European study though further research on ABI in Ireland is required in order to effectively plan, resource and deliver services needed by such persons and their families.

## Competing interests

The authors declare that they have no competing interests.

## Authors' contributions

FF is a medical student researcher. She collected and analysed the data and led the write up of this paper with AMacF. LG and BD contributed to the design of the study, data analysis and write up. FC contributed to the study design and write up. AMacF is Principal Investigator, research supervisor for FF. She contributed to the study design, data collection and write up. All authors have read and approved the final manuscript.

## Pre-publication history

The pre-publication history for this paper can be accessed here:



## Supplementary Material

Additional file 1 **Questionnaire for postal survey**. Questionnaire used for postal survey of general practitioners on prevalence of ABI. Click here for file
