# Supplementary material for: A postal survey of data in general practice on the prevalence of Acquired Brain Injury (ABI) in patients aged 18–65 in one county in the west of Ireland
Source: BMC Fam Pract. 2009 May 19;10:36. doi: 10.1186/1471-2296-10-36 (PMC2695423; doi:10.1186/1471-2296-10-36)
Supplement: Additional file 1 — Questionnaire for postal survey. Questionnaire used for postal survey of general practitioners on prevalence of ABI. [file 1471-2296-10-36-S1.doc]

**G.P. Questionnaire on Prevalence of Acquired Brain Injury (ABI) in people aged 18-65 in Mayo, Galway and Roscommon.**

Practice Name: _______________________________________________________

Number of G.P’s working in practice: ______________________________________

Approximate total practice patient population: _______________________________

Are you aware of any patients in your practice with Acquired Brain Injury between the ages of 18-65? *(Please tick appropriate box)*

□ **Yes □ No □ Don’t know for certain**

**If yes**, approximately how many patients?

_____________________________________________________________________

- **If yes**, Please complete the following grid as much as possible

| Patient Age | Patient Gender | Age at Diagnosis | Type Of ABI |
| --- | --- | --- | --- |
|  |  |  |  |
|  |  |  |  |
|  |  |  |  |
|  |  |  |  |
|  |  |  |  |
|  |  |  |  |
|  |  |  |  |
|  |  |  |  |
|  |  |  |  |

**Types of ABI :**

- Traumatic Brain Injury e.g. RTA, accidents, assault, falls
- Brain Tumour
- Brain Haemmorhage
- Brain Infection e.g. Encephalitis/Meningitis
- Hypoxic Brain Injury e.g Near Drowning, Attempted Suicide
